# Supplementary material for: α-Rank: Multi-Agent Evaluation by Evolution
Source: Sci Rep. 2019 Jul 9;9:9937. doi: 10.1038/s41598-019-45619-9 (PMC6617105; doi:10.1038/s41598-019-45619-9)
Supplement: Supplementary file 1 — supplementary material [file 41598_2019_45619_MOESM1_ESM.pdf]

α-Rank: Multi-Agent Evaluation by Evolution (Supplementary Material)

Shayegan Omidshafiei*, Christos H. Papadimitriou*, Georgios Piliouras*, Karl Tuyls*, Mark Rowland, Jean-Baptiste Lespiau, Wojciech M. Czarnecki, Marc Lanctot, Julien Perolat, Remi Munos
*Equal contributors, ordered alphabetically. Corresponding author: Karl Tuyls <karltuyls@google.com>.

S1 Most Closely Related Work

We describe related work revolving around Empirical Game Theory analysis (EGTA), discrete-time dynamics models and multi-agent interactions in evolution of cooperation research, and precursors to our new solution concept of MCC.

The purpose of the first applications of EGTA was to reduce the complexity of large economic problems in electronic commerce, such as continuous double auctions, supply chain management, market games, and automated trading [21, 46, 49, 50, 52]. While these complex economic problems continue to be a primary application area of these methods [7, 47, 48, 51], the general techniques have been applied in many different settings. These include analysis of interactions among heuristic meta-strategies in poker [32], network protocol compliance [53], collision avoidance in robotics [15], and security games [25, 33, 54].

Evolutionary dynamics have often been presented as a practical tool for analyzing interactions among meta-strategies found in EGTA [6, 15, 49], and for studying the change in policies of multiple learning agents [6], as the EGTA approach is largely based on the same assumptions as evolutionary game-theory, viz. repeated interactions among sub-groups sampled independently at random from an arbitrarily-large population of agents.

From the theoretical biology perspective, researchers have additionally deployed discrete-time evolutionary dynamics models [26]. These models typically provide insights in the macro-dynamics of the overall behavior of agents in strategy space, corresponding to flow rates at the edges of a manifold [27, 35, 36, 42, 43]. These studies usually focus on biological games, the evolution of cooperation and fairness in social dilemma's like the iterated prisoner's dilemma or signalling games, deploying, amongst others, imitation dynamics with low mutation rates [11, 45]. Similar efforts investigating evolutionary dynamics inspired by statistical physics models have been taken as well [22, 39].

In the framework of *the evolution of conventions* [55], a repeated game is played by one-time players who learn from past plays and are subject to noise and mistakes. Essentially algorithmic, and in the same line of thought as our formalism, it solves the equilibrium selection problem of *weakly acyclic* games (in our terminology explained in Section 2.4.3: games whose sink strongly connected components happen to be singletons), and in this special case it aligns very well with our proposed solution concept. Another equilibrium selection concept related to MCC is the concept of *closed under rational behavior (CURB)* set of strategies [5]. The notion of a *sink equilibrium*, defined by [13] for the purpose of exploring new variants of the price of anarchy, is also similar to our MCC — despite differences in mathematical detail, style, and use. Conley's index theory has also been studied in detail in dynamical systems research, e.g. see [1]. A method for decomposing games was introduced in [8], based on properties of game dynamics on the graph of pure strategy profiles by exploiting conceptually similarities to the structure of continuous vector fields. Researchers have also carried out studies of evaluation metrics in the fields of computer science, machine learning, and artificial intelligence [16, 17, 20]. PageRank [28], an algorithm used for ranking webpages, uses a Markov chain where states are webpages and transitions capture links between these pages; though the Markov chain foundations are related to those used in our work, they are not rooted in an evolutionary dynamical system nor in a game-theoretic solution concept, and as such are quite different to the method presented here which also generalizes across several dimensions. The Elo rating system has ubiquitously been used for ranking and predicting outcomes in board games [9], sports [2, 10, 19, 23, 38], and artificial intelligence [24, 37]. This rating system, however, comes with two key limitations [4]: first, it has no predictive power in games with intransitive (cyclic) relations in the set of evaluated agents (e.g., in Rock-Paper-Scissors); second, the rating of a given agent can be artificially inflated by including duplicate copies of weaker agents in the set.

S2 Background in Dynamical Systems

This section introduces the technical background necessary for understanding the paper contribution. Parts of this section involve mathematical preliminaries discussed in more detail in [29].

Definition S2.0.1 (Map). Let X be a set. A *map* is a function $f : X \to X$.

Definition S2.0.2 (Flow). A *flow* $\phi : \mathbb{R} \times X \to X$ on a topological space X is a continuous map that satisfies the following properties:

(i) For all $x \in X$, $\phi(0,x) = x$.

(ii) For each $t \in \mathbb{R}$, $\phi(t,\cdot) : X \to X$ is a homeomorphism.

(iii) For all $s,t \in \mathbb{R}$ and all $x \in X$. $\phi(s+t,x) = \phi(s,\phi(t,x))$.

Sometimes it is convenient to write $\phi^t(x)$ for $\phi(t,x)$ and denote a flow $\phi : \mathbb{R}\times X \to X$ by $\phi^t : X \to X$. We also use the notation ϕ^t at times to denote map $\phi(t,\cdot)$ for a fixed t. The map ϕ^1 is used to relate the behavior of a flow to the behavior of a (discrete-time) map.

Definition S2.0.3 (Time-one map). Given flow $\phi(t,\cdot)$ the function ϕ^1 defines the time-one map of ϕ.

The dynamical systems that we consider are well behaved. For example, the state space of replicator dynamics is compact and its vector field is Lipschitz-continuous, so we we can represent its unique solution by a flow. Given an initial condition $x \in X$, this defines a function of time that expresses the system's orbit or trajectory for that starting position. If the starting point is not a fixed point (i.e. equilibrium) then we wish to understand its long-term behavior. (informally the limit of $\phi(t,x)$ when t goes to infinity). Since in many cases such functions do not exhibit a unique limit point we need to understand the limit behavior of all possible convergent subsequences. Concretely, given an initial condition $x \in X$, we call point $y \in X$ an ω-limit point of the trajectory through x if there exists a sequence $(t_i)_{i\in\mathbb{N}} \in \mathbb{R}$ such that $\lim_{i\to\infty} t_i = \infty$, $\lim_{i\to\infty}\phi(t_i,x) = y$.

Definition S2.0.4 (Homeomorphism). A function g between two topological spaces is called a *homeomorphism* if it is a continuous bijection, and has a continuous inverse.

Liouville's Theorem Let $g = g(x)$, $g : \mathbb{R}^d \to \mathbb{R}^d$ be a vector field. The divergence of g is is defined as the trace of the corresponding Jacobian at x, *i.e.*, $\mathrm{div}[g(x)] = \sum_{i=1}^{d} \frac{\partial g_i}{\partial x_i}(x)$. A 'divergence free' vector field has zero divergence everywhere. A key property of divergence-free vector fields is that their associated flow maps are volume preserving.

Theorem S2.0.5 (Liouville's Theorem). Let f be a divergence free vector field. Then its flow map ϕ^t is volume preserving (for all t).

S2.1 Multi-population Replicator Dynamics

For a K-player NFG, one may use a set of K populations with S^k denoting the finite set of pure strategies available to each agent in population $k \in \{1,\dots,K\}$. The mass of players in a given population k that use strategy $i \in S^k$ is denoted x_i^k, where $\sum_{i\in S^k} x_i^k = 1$. Let S denote the set of all populations' pure strategy profiles, and x represent the joint population state. Let the payoff matrix for a given population k be denoted $M^k : S \to \mathbb{R}$. The fitness of an agent in population k playing pure strategy i given state x is then,

$$f_i^k(x) = \sum_{s^{-k}\in S^{-k}} M^k(i,s^{-k}) \prod_{c\neq k} x^c_{s^c}. \tag{1}$$

Namely, the fitness is the expected payoff the agent playing strategy i receives given every competitor population's state x^c. The k-th population's average fitness given state x is then,

$$\bar{f}^k(x) = \sum_i f_i^k(x) x_i^k, \tag{2}$$

with the corresponding K-population replicator dynamics,

$$\dot{x}_i^k = x_i^k(f_i^k(x) - \bar{f}^k(x)) \qquad \forall k \in \{1,\dots,K\} \quad \forall i \in s^k. \tag{3}$$

S2.2 Single-population discrete-time model

We have a set of strategies (or agents under evaluation) $S = \{s_1,..,s_n\}$, with $|S| = n$, which we would like to evaluate for their evolutionary strength. We also have a population of individuals $A = \{a_1,..,a_m\}$, with $|A| = m$, that are programmed to play a strategy from the set S. Individuals interact pairwise through empirical games.

We start from a finite well-mixed population of m individuals, in which p individuals are playing τ. At each timestep t we randomly choose two individuals τ and σ, with respective strategies s_τ and s_σ. The strategy of individual τ is then updated by either probabilistically copying the strategy s_σ of individual σ it is interacting with, mutating with a very small probability into another strategy, or sticking with its own strategy s_τ. The idea is that strong individuals will replicate and spread throughout the population. The probability with which individual τ (playing s_τ) will copy strategy s_σ from individual σ can be described by a *selection* function $\mathbb{P}(\tau \to \sigma)$, which governs the dynamics of the finite-population model.

Individual τ will thus copy the behavior of individual σ with probability $p_{\tau\to\sigma}$ and stick to its own strategy with probability $1-\mathbb{P}(\tau\to\sigma)$. We denote the probability for a strategy to mutate randomly into another strategy $s \in S$ by μ and we will assume it to be infinitesimally small, (i.e., we consider a small-mutation limit $\mu \to 0$). If we neglected mutations, the end state of this evolutionary process would be monomorphic. If we introduce a very small mutation rate this means that either the mutant fixates and takes over the current population, or the current population is capable of wiping out the mutant strategy [11]. Therefore, given a small mutation rate, the mutant either fixates or disappears before a new mutant appears. This means that the population will never contain more than two strategies at any point in time.

We now proceed as follows. At any moment in time when two strategies (s_τ and s_s) are present in the population, we can calculate the fitness of an individual τ playing strategy s_τ in a population of p individuals playing s_τ and $m-p$ individuals playing s_s. Fitnesses may be calculated using either knowledge of the global population state (i.e., where every individual is aware of the number of other individuals playing each strategy, which may be a strong assumption) or local knowledge (i.e., only the current opponent's strategy) [41]. The corresponding fitness for the local case, which we focus on here, is $f(\tau,\sigma) = M_{\tau,\sigma}$, where $M_{\tau,\sigma}$ is obtained from the meta-game payoff matrix. Analogously, the simultaneous payoff of an individual σ playing s_σ against s_τ is $f(\sigma,\tau) = M_{\sigma,\tau}$, For the remainder of the paper, we focus on the logistic selection function (aka Fermi distribution),

$$\mathbb{P}(\tau \to \sigma) = \frac{e^{\alpha f(\sigma,\tau)}}{e^{\alpha f(\tau,\sigma)} + e^{\alpha f(\sigma,\tau)}} = (1 + e^{\alpha(f(\tau,\sigma) - f(\sigma,\tau))})^{-1}, \tag{4}$$

with α determining the selection intensity. While the subsequent empirical methodology extends to general selection functions, the choice of Fermi selection function enables closed-form characterization of certain properties of the discrete-time model.

Based on this setup, we define a Markov chain over the set of strategies S with n states. Each state represents a monomorphic population end-state, corresponding to one of the strategies s_τ with $\tau \in \{1,..,n\}$. The transitions between these states are defined by the corresponding fixation probabilities when a mutant strategy is introduced in a monomorphic population. The stationary distribution over this Markov chain will tell us how much time on average the dynamics will spend in each of the monomorphic states.

Considering our set S of n strategies, we define the Markov chain with n^2 transition probabilities over the monomorphic states. Let $\eta = \frac{1}{n-1}$ and denote by $\rho_{\sigma,\tau}$ the probability of mutant strategy s_τ fixating (taking over) in a resident population of individuals playing s_σ. So $\eta\rho_{\sigma,\tau}$ is the probability that a population which finds itself in state s_σ will end up in state s_τ after the occurrence of a single mutation. This yields the following Markov transition matrix,

$$C = \begin{pmatrix} 1-\eta(\rho_{1,2}+\rho_{1,3}+\ldots+\rho_{1,n}) & \eta\rho_{1,2} & \ldots & \eta\rho_{1,n} \\ \eta\rho_{2,1} & 1-\eta(\rho_{2,1}+\rho_{2,3}+\ldots+\rho_{2,n}) & \ldots & \eta\rho_{2,n} \\ \ldots & \ldots & \ldots & \ldots \\ \eta\rho_{n,1} & \ldots & \ldots & 1-\eta(\rho_{n,1}+\rho_{n,2}+\ldots+\rho_{n,n-1}) \end{pmatrix} \tag{5}$$

The fixation probabilities $\rho_{\sigma,\tau}$ can be calculated as follows. Assume we have a population of p individuals playing s_τ and $m-p$ individuals playing s_σ. The probability that the number of type s_τ individuals decreases/increases by one is given by,

$$T^{(\mp 1)}(p,\tau,\sigma) = \frac{p(m-p)}{m(m-1)}\left(1 + e^{\pm\alpha(f(\tau,\sigma)-f(\sigma,\tau))}\right)^{-1}. \tag{6}$$

Now we can compute the fixation probability $\rho_{\sigma,\tau}$ of a mutant with strategy s_τ in a population of $m-1$ individuals programmed to playing s_σ. Specifically, let $u = f(\tau,\sigma) - f(\sigma,\tau)$. Then,

$$\rho_{\sigma,\tau} = \left(1 + \sum_{l=1}^{m-1}\prod_{p=1}^{l}\frac{T^{(-1)}(p,\tau,\sigma)}{T^{(+1)}(p,\tau,\sigma)}\right)^{-1} \tag{7}$$

$$= \left(1 + \sum_{l=1}^{m-1}\prod_{p=1}^{l}\frac{(1+e^{\alpha u})^{-1}}{(1+e^{-\alpha u})^{-1}}\right)^{-1} \tag{8}$$

$$= \left(1 + \sum_{l=1}^{m-1}\prod_{p=1}^{l}\frac{1+e^{-\alpha u}}{1+e^{\alpha u}}\right)^{-1} \tag{9}$$

$$= \left(1 + \sum_{l=1}^{m-1}\prod_{p=1}^{l}\frac{\frac{e^{\alpha u}+1}{e^{\alpha u}}}{1+e^{\alpha u}}\right)^{-1} \tag{10}$$

$$= \left(1 + \sum_{l=1}^{m-1}\prod_{p=1}^{l} e^{-\alpha u}\right)^{-1} \tag{11}$$

$$= \left(1 + \sum_{l=1}^{m-1}\prod_{p=1}^{l} e^{-\alpha(f(\tau,\sigma)-f(\sigma,\tau))}\right)^{-1} \tag{12}$$

This corresponds to the computation of an m-step transition in the Markov chain [40].The quotient $\frac{T^{(-1)}(p,\tau,\sigma)}{T^{(+1)}(p,\tau,\sigma)}$ expresses the likelihood (odds) that the mutation process continues in either direction: if it is close to zero then it is very likely that the

number of mutants s_τ increases; if it is very large it is very likely that the number of mutants will decrease; and if it close to one then the probabilities of increase and decrease of the number of mutants are equally likely.

Property S2.2.1. Given finite payoffs, fixation probabilities $\rho_{\sigma,\tau}$ under the Fermi imitative protocol (4) are positive for all σ and τ; i.e., any single mutation can cause a transition from any state to another. Markov chain C is, therefore, irreducible, and a unique stationary distribution π (where $\pi^T C = \pi^T$ and $\sum_i \pi_i = 1$) exists.

This unique π provides the evolutionary ranking, or strength of each strategy in the set S, expressed as the time the population spends in each state in distribution π. This single population model has been widely studied (see, e.g., [27, 35, 36, 42, 43]), both theoretically and empirically, but is limited to both pairwise interactions and symmetric games.

A note on the correctness of the small-mutation assumption: We have noted that in a stochastic system, such as the one we consider here, in the limit of mutation rate $\mu = 0$ one always fixates in a monomorphic population through drift (even if it may take some time for this fixation to occur). The assumption of a small enough mutation rate to make the monomorphic state analysis reasonably accurate is a practical one, necessary for enabling the tractible analysis of finite-population interactions. This assumption has been made in numerous related works [12, 14, 18, 35]. Indeed, to include the time the system spends in co-existence states (e.g., in games where the evolutionarily stable strategy involves the population spending a non-negligible amount of time in a non-monomorphic state), one would need to add a correction in the model; this holds even in the case of a single population playing a symmetric game, e.g., as considered in recent works such as [44]. However, this line of analysis is considered beyond the scope of this paper and is left as future work.

S3 Proofs

S3.1 Proof of Theorem 2.1.2

Theorem 2.1.2. Given finite payoffs, the Markov chain with transition matrix C is irreducible (i.e., it is possible to get to any state starting from any state). Thus a unique stationary distribution π (where $\pi^T C = \pi^T$ and $\sum_i \pi_i = 1$) exists.

Proof. Consider any two states (i.e., strategy profiles) s_i and s_j of the K-population Markov chain with transition matrix (14). Under finite payoffs $f^k(\tau, p)$ and $f^k(\sigma, p)$, fixation probabilities $\rho^k_{\sigma,\tau}$ under the Fermi imitative protocol (4) are positive. Let $R(s,k,s_x)$ denote the operation of replacing the k-th strategy in a strategy profile s with a different pure strategy s_x. Thus, state s_j is accessible from any state s_i (namely, consider the chain $\{s_0 = s_i, s_1 = R(s_0, 1, s_j^1), s_2 = R(s_1, 2, s_j^2) \ldots, s_{K-1} = R(s_{K-2}, K-1, s_j^{K-1}), s_K = R(s_{K-1}, K, s_j^K) = s_j\}$ connecting strategies s_i to s_j with non-zero probability). The Markov chain is, therefore, irreducible and a unique stationary distribution exists. □

S3.2 Proof for Theorem 2.1.4

Theorem 2.1.4 (Discrete-Continuous Edge Dynamics Correspondence). In the large-population limit, the macro-dynamics model is equivalent to the micro-dynamics model over the edges of the strategy simplex. Specifically, the limiting model is a variant of the replicator dynamics with the caveat that the Fermi revision function takes the place of the usual fitness terms.

Proof. To simplify notation, we prove the theorem for the single-population case without loss of generality. Let $x_i(t)$ represent the fraction of individuals in the population that are playing strategy s_i at timestep t. Rather than consider the underlying stochastic evolutionary equations directly, we consider the *mean dynamics*. An alternative proof path for the single population case is presented in [41] and may be applied here as well. The mean dynamics constitute a deterministic process governing the expected evolution of state $x_i(t)$, and provide a close approximation of the underlying system over finite time spans under a large-population limit [34, Chapters 4 and 10]. For a general finite population game, the mean dynamics correspond to the difference of the expected influx and outflux of individuals playing a strategy i against individuals playing any strategy $j \in S$ given the underlying selection function $\mathbb{P}(i \to j)(x)$,

$$\dot{x}_i(t) = \sum_{j \in S} x_j x_i \mathbb{P}(j \to i)(x) - x_i \sum_{j \in S} x_j \mathbb{P}(i \to j)(x). \tag{13}$$

Under the low-mutation rate assumption, the finite population model considers only the transitions between pairs of monomorphic states s_τ and s_σ, where $x_\tau + x_\sigma = 1$. This yields simplified mean dynamics,

$$\begin{aligned}
\dot{x}_\tau &= x_\sigma x_\tau \mathbb{P}(\sigma \to \tau) - x_\tau x_\sigma \mathbb{P}(\tau \to \sigma)(x_\tau) && (14)\\
&= (1 - x_\tau) x_\tau \left[\mathbb{P}(\sigma \to \tau) - \mathbb{P}(\tau \to \sigma)\right] && (15)\\
&= x_\tau \left[\mathbb{P}(\sigma \to \tau) - (x_\tau \mathbb{P}(\sigma \to \tau) + (1 - x_\tau)\mathbb{P}(\tau \to \sigma))\right] && (16)\\
&= x_\tau \left[\mathbb{P}(\sigma \to \tau) - \bar{p}\right] && (17)
\end{aligned}$$

where,

$$\mathbb{P}(\tau \to \sigma) = (1+e^{\alpha(f(\tau,\sigma)-f(\sigma,\tau))})^{-1} \tag{18}$$

$$\mathbb{P}(\sigma \to \tau) = (1+e^{-\alpha(f(\tau,\sigma)-f(\sigma,\tau))})^{-1} \tag{19}$$

$$\bar{p} = x_\tau \mathbb{P}(\sigma \to \tau) + (1-x_\tau)\mathbb{P}(\tau \to \sigma) \tag{20}$$

We, therefore, observe that the discrete large-population mean dynamics (17) correspond to the replicator equations (with the caveat that Fermi revision protocol takes the place of the usual fitness terms).

Moreover, one can branch off after (15) to yield,

$$\dot{x}_\tau = (1-x_\tau)x_\tau\left[\mathbb{P}(\sigma \to \tau) - \mathbb{P}(\tau \to \sigma)\right] \tag{21}$$

$$= x_\tau(1-x_\tau)\left[\left(1+e^{-\alpha(f(\tau,\sigma)-f(\sigma,\tau))}\right)^{-1} - \left(1+e^{\alpha(f(\tau,\sigma)-f(\sigma,\tau))}\right)^{-1}\right] \tag{22}$$

$$= x_\tau(1-x_\tau)\tanh\frac{\alpha(f(\tau,\sigma)-f(\sigma,\tau))}{2} \tag{23}$$

which matches the Itô calculus based derivation of [42] under the large-population limit. □

S3.3 Proof for Theorem 2.4.12

We start by introducing the notion of chain transitivity, which will be useful in the proof of the theorem.

Definition S3.3.1 (Chain transitive). Let ϕ be a flow on a metric space (X,d). A set $A \subset X$ is chain transitive with respect to ϕ if for any $x,y \in A$ and any $\varepsilon > 0$ and $T > 0$ there exists an (ε,T)-chain from x to y.

Next we state the following properties of chain components,

Property S3.3.2 ([3]). Each chain component of a flow on a compact metric space is closed, connected, and invariant with respect to the flow. Also,

- Each chain component of a flow on a metric space is chain transitive with respect to the flow.
- Every chain transitive set with respect to a flow on a metric space is a subset of a unique chain component of the flow.
- If A and B are chain transitive with respect to a flow on a metric space, $A \subset B$ and C is the unique chain component containing A, then $B \subset C$.

Theorem 2.4.12 (Partial order on chain components). Let ϕ be a flow on a metric space and A_1, A_2 be chain components of the flow. Then the relation defined by $A_1 \leq_C A_2$ is a partial order.

Proof. We will show that the binary relation $\leq_C$ is reflective, antisymmetric and transitive.

- $A_1 \leq_C A_1$. Since any chain component is chain transitive then we have that for any $x,y \in A_1$: $x \sim y$.
- If $A_1 \leq_C A_2$ and $A_2 \leq_C A_1$ then $A_1 = A_2$. By chain transitivity of A_1, A_2 we have that for any $x,x' \in A_1$, $x \sim x'$ and for any $y,y' \in A_2$, $y \sim y'$. Hence if $x \sim y$ then $x' \sim y'$ for any $x' \in A_1$ and any $y' \in A_2$. Hence, $A_1 \cup A_2$ is a chain transitive set and thus by Theorem S3.3.2 must be a subset of a unique chain component of the flow C such that $A_1 \cup A_2 \subset C$. However, we assumed that A_1, A_2 are chain components themselves. Thus, $A_1 = A_1 \cup A_2 = A_2$.
- If $A_1 \leq_C A_2$ and $A_2 \leq_C A_3$ then $A_1 \leq_C A_3$. If there exist $x \in A_1$ and $y \in A_2$ such that $x \in \Omega^+(\phi,y)$, as well as $y' \in A_2$ and $z \in A_3$ such that $y' \in \Omega^+(\phi,z)$ then by chain transitivity of A_2 we have that $y \in \Omega^+(\phi,y')$ and thus $x \in \Omega^+(\phi,z)$, implying $A_1 \leq_C A_3$.

□

S3.4 Proof for Theorem 2.4.23

We first present several results necessary for the proof.

Lemma S3.4.1. A chain recurrent (CR) point x is a sink CR point if and only if for any CR point y if $y \in \Omega^+(\phi,x)$ then $x \in \Omega^+(\phi,y)$, i.e., the two points are chain equivalent.

Proof. We will argue the forward direction by contradiction. Suppose not. That is, suppose that x is a sink CR point and there exists a CR point y such that $y \in \Omega^+(\phi,x)$ and $x \notin \Omega^+(\phi,y)$, then if C_x, C_y are the equivalence classes/chain components of x,y respectively we have that $C_y \leq_C C_x$ and C_y, C_x are clearly distinct chain components since $x \notin \Omega^+(\phi,y)$. Thus, C_x is not a sink chain component and x is not a sink chain recurrent point, contradiction.

For the reverse direction, once again by contradiction we have that for any CR point y with $y \in \Omega^+(\phi, x)$, $x \in \Omega^+(\phi, y)$ and x is a non-sink CR point. Then there exists another chain component A with $A \leq_C C_x$ where C_x is the equivalence class/chain component of x. Hence, there exists $y \in A$ such that $y \in \Omega^+(\phi, x)$. Since y is a CR point which does not belong to C_x, we have $x \notin \Omega^+(\phi, y)$, contradiction. □

Lemma S3.4.2. If a sink chain component contains a single vertex s_i then it contains any vertex s_j which is reachable from s_i via (weakly)-better response moves. Specifically, it contains an MCC.

Proof. Any state/vertex s_j is a chain recurrent (CR) point because it is a fixed point of the replicator dynamics. If s_j is reachable by s_i via a weakly-better response path, then $s_j \in \Omega^+(\phi, s_i)$ for the replicator flow. In the case of edges that are strictly improving it suffices to use the ε correction to introduce the improving strategy and replicator will converge to the better outcome. In the case of edges between outcomes of equal payoff all convex combinations of these strategies are fixed points for the replicator and we can traverse this edge with $\lceil 1/\varepsilon \rceil$ hops of size ε.

But if $s_j \in \Omega^+(\phi, s_i)$ and s_i is a sink CR point (since it belongs to a CR component) then by Lemma S3.4.1 $s_i \in \Omega^+(\phi, s_j)$. Therefore, state/vertex s_j also belongs to the same sink chain component. The set of reachable vertices includes a strongly connected component with no outgoing edges and thus a MCC.

□

Theorem 2.4.23. Let ϕ be the replicator flow when applied to a K-player game. The number of asymptotically stable sink chain components is finite. Specifically, every asymptotically stable sink chain component contains at least one MCC; each MCC is contained in exactly one chain component.

Proof. Since solutions in the neighborhood of an asymptotically stable set, all approach the set, volume is contracted in this neighborhood, however, replicator dynamics is volume preserving in the interior of the state space [30, 31, 34]; the formal argument, presented in detail in these references, works by transforming the system induces by the replicator dynamics over the interior of the state space into a conjugate dynamical system that is divergence-free.

Any asymptotically stable set cannot lie in the interior of the simplex, i.e., it cannot consist only of fully mixed strategies. Hence, there must exist some product of subsimplices with a non-empty intersection with this set. The intersection of the original asymptotically stable set with this subspace is still asymptotically stable for this invariant subspace and thus we can continue the argument inductively. The intersection of the attracting neighborhood with this subspace is an attracting neighborhood for the dynamics on this invariant subspace. We deduce that any asymptotically stable chain component must contain at least one vertex of the simplex (pure strategy profile). Let s_i be this vertex. By Lemma S3.4.2, this sink chain component must also include all other vertices reachable from s_i via weakly-better replies. Specifically, it must include at least one MCC. Finally, a MCC is a chain transitive set for the replicator flow via the same argument of the ε hops as in Lemma S3.4.2. By Theorem S3.3.2, it is a subset of a unique chain component of the flow. □

S3.5 Proof for Theorem 2.5.1

Theorem 2.5.1. In the limit of infinite ranking-intensity α, the Markov chain associated with the generalized multi-population model introduced in Section 2.1.4 coincides with the MCC.

Proof. Recall from the MCC definition that the probability of strictly improving responses for all players are set equal to each other, and transitions between strategies of equal payoff happen with a smaller probability also equal to each other for all players. Let the ratio of the two probabilities be denoted ε for all players. The transition probabilities of the Markov chain of the macro-model when taking taking the limit of $\alpha \to \infty$ are equal to the transitions probabilities of the Markov chain of the Markov-Conley chains when setting ε equal to $\frac{1}{m}$, where m is the size of the population in the macro-model. Let $A_{s_i}(k)$ be the number of strictly improving moves for player k in state/vertex s_i. Similarly, let $B_{s_i}(k)$ be the number of deviating moves for player k in state/vertex s_i that do not affect her payoff. It suffices to set the probability of a node s_i self-transitioning equal to $1 - \frac{\sum_k A_{s_i}(k) + \varepsilon \sum_k B_{s_i}(k)}{\sum_k (|S^k| - 1)}$. □

References

1. A natural order in dynamical systems based on conley–markov matrices. *Journal of Differential Equations*, 252(4):3116 – 3141, 2012.

2. David Aldous. Elo ratings and the sports model: A neglected topic in applied probability? *Statist. Sci.*, 32(4):616–629, 11 2017. doi: 10.1214/17-STS628.

3. John M Alongi and Gail Susan Nelson. *Recurrence and Topology*, volume 85. American Mathematical Soc., 2007.

4. David Balduzzi, Karl Tuyls, Julien Perolat, and Thore Graepel. Re-evaluating Evaluation. *arXiv*, (1806.02643), 2018.

5. Kaushik Basu and Jörgen W Weibull. Strategy subsets closed under rational behavior. *Economics Letters*, 36(2):141–146, 1991.

6. Daan Bloembergen, Karl Tuyls, Daniel Hennes, and Michael Kaisers. Evolutionary dynamics of multi-agent learning: A survey. *J. Artif. Intell. Res. (JAIR)*, 53:659–697, 2015.

7. E. Brinkman and M.P. Wellman. Shading and efficiency in limit-order markets. In *Proceedings of the IJCAI-16 Workshop on Algorithmic Game Theory*, 2016.

8. Ozan Candogan, Ishai Menache, Asuman Ozdaglar, and Pablo A Parrilo. Flows and decompositions of games: Harmonic and potential games. *Mathematics of Operations Research*, 36(3):474–503, 2011.

9. Arpad E. Elo. *The Rating of Chess players, Past and Present*. Ishi Press International, 1978.

10. Wunderlich F and Memmert D. The Betting Odds Rating System: Using soccer forecasts to forecast soccer. *PLoS ONE*, 6 (13):e0198668, 2018.

11. Drew Fudenberg and Lorens A Imhof. Imitation processes with small mutations. *Journal of Economic Theory*, 131(1): 251–262, 2006.

12. Julián García and Arne Traulsen. The structure of mutations and the evolution of cooperation. *PloS one*, 7(4):e35287, 2012.

13. Michel Goemans, Vahab Mirrokni, and Adrian Vetta. Sink equilibria and convergence. In *Foundations of Computer Science, 2005. FOCS 2005. 46th Annual IEEE Symposium on*, pages 142–151. IEEE, 2005.

14. Christoph Hauert, Arne Traulsen, Hannelore Brandt, Martin A Nowak, and Karl Sigmund. Via freedom to coercion: the emergence of costly punishment. *science*, 316(5833):1905–1907, 2007.

15. D. Hennes, D. Claes, and K Tuyls. Evolutionary advantage of reciprocity in collision avoidance. In *Proceedings of the AAMAS 2013 Workshop on Autonomous Robots andMultirobot Systems (ARMS 2013)*, 2013.

16. José Hernández-Orallo. Evaluation in artificial intelligence: from task-oriented to ability-oriented measurement. *Artificial Intelligence Review*, 48(3):397–447, 2017.

17. José Hernández-Orallo. *The measure of all minds: evaluating natural and artificial intelligence*. Cambridge University Press, 2017.

18. Christian Hilbe, Martin A Nowak, and Karl Sigmund. Evolution of extortion in iterated prisoner's dilemma games. *Proceedings of the National Academy of Sciences*, 110(17):6913–6918, 2013.

19. Lars Magnus Hvattum and Halvard Arntzen. Using ELO ratings for match result prediction in association football. *International Journal of Forecasting*, 26(3):460 – 470, 2010. Sports Forecasting.

20. Nathalie Japkowicz and Mohak Shah. *Evaluating learning algorithms: a classification perspective*. Cambridge University Press, 2011.

21. Christopher Kiekintveld and Michael P. Wellman. Selecting strategies using empirical game models: an experimental analysis of meta-strategies. In *7th International Joint Conference on Autonomous Agents and Multiagent Systems (AAMAS 2008), Estoril, Portugal, May 12-16, 2008, Volume 2*, pages 1095–1101, 2008.

22. Linjie Liu, Shengxian Wang, Xiaojie Chen, and Matjaž Perc. Evolutionary dynamics in the public goods games with switching between punishment and exclusion. *Chaos*, 28(1):103105, 2018.

23. Cattelan Manuela, Varin Cristiano, and Firth David. Dynamic Bradley–Terry modelling of sports tournaments. *Journal of the Royal Statistical Society: Series C (Applied Statistics)*, 62(1):135–150, 2013.

24. Volodymyr Mnih, Koray Kavukcuoglu, David Silver, Andrei A. Rusu, Joel Veness, Marc G. Bellemare, Alex Graves, Martin Riedmiller, Andreas K. Fidjeland, Georg Ostrovski, Stig Petersen, Charles Beattie, Amir Sadik, Ioannis Antonoglou, Helen King, Dharshan Kumaran, Daan Wierstra, Shane Legg, and Demis Hassabis. Human-level control through deep reinforcement learning. *Nature*, 518(7540):529–533, 02 2015.

25. T.H. Nguyen, M. Wright, M.P. Wellman, and S. Singh. Multi-stage attack graph security games: Heuristic strategies, with empirical game-theoretic analysis. In *Proceedings of the Fourth ACM Workshop on Moving Target Defense*, 2017.

26. M. A. Nowak. *Evolutionary Dynamics: Exploring the Equations of Life*. Harvard University Press, 2006.

27. Martin A. Nowak and Karl Sigmund. Evolutionary dynamics of biological games. *Science*, 303(5659):793–799, 2004.

28. Lawrence Page, Sergey Brin, Rajeev Motwani, and Terry Winograd. The pagerank citation ranking: Bringing order to the web. Technical report, Stanford InfoLab, 1999.

29. Christos Papadimitriou and Georgios Piliouras. From Nash equilibria to chain recurrent sets: Solution concepts and topology. In *Proceedings of the 2016 ACM Conference on Innovations in Theoretical Computer Science*, ITCS '16, pages 227–235, New York, NY, USA, 2016. ACM. ISBN 978-1-4503-4057-1.

30. G. Piliouras and J. S. Shamma. Optimization despite chaos: Convex relaxations to complex limit sets via Poincaré recurrence. In *Symposium of Discrete Algorithms (SODA)*, 2014.

31. Georgios Piliouras, Carlos Nieto-Granda, Henrik I. Christensen, and Jeff S. Shamma. Persistent patterns: Multi-agent learning beyond equilibrium and utility. In *AAMAS*, pages 181–188, 2014.

32. Marc J. V. Ponsen, Karl Tuyls, Michael Kaisers, and Jan Ramon. An evolutionary game-theoretic analysis of poker strategies. *Entertainment Computing*, 1(1):39–45, 2009.

33. A Prakash and M.P. Wellman. Empirical game-theoretic analysis for moving target defense. In *Proceedings of the Second ACM Workshop on Moving Target Defense*, 2015.

34. W.H. Sandholm. *Population Games and Evolutionary Dynamics*. Economic Learning and Social Evolution. MIT Press, 2010. ISBN 9780262288613.

35. Francisco C. Santos, Jorge M. Pacheco, and Brian Skyrms. Co-evolution of pre-play signaling and cooperation. *Journal of Theoretical Biology*, 274(1):30–35, 2011.

36. Sven Van Segbroeck, Jorge M. Pacheco, Tom Lenaerts, and Francisco C. Santos. Emergence of fairness in repeated group interactions. *Physical Review Letters*, 108:158104, 2012.

37. David Silver, Thomas Hubert, Julian Schrittwieser, Ioannis Antonoglou, Matthew Lai, Arthur Guez, Marc Lanctot, Laurent Sifre, Dharshan Kumaran, Thore Graepel, et al. A general reinforcement learning algorithm that masters chess, shogi, and go through self-play. *Science*, 362(6419):1140–1144, 2018.

38. Connor Sullivan and Christopher Cronin. Improving Elo rankings for sports experimenting on the english premier league. In *Virginia Tech CSx824/ECEx424 technical report*, 2016.

39. Attila Szolnoki and Matjaz Perc. Evolutionary dynamics of cooperation in neutral populations. *New Journal of Physics*, 20 (1):013031, 2018.

40. Howard M. Taylor and Samuel Karlin. *An Introduction To Stochastic Modeling*. Academic Press, third edition edition, 1998.

41. Arne Traulsen, Jens Christian Claussen, and Christoph Hauert. Coevolutionary dynamics: from finite to infinite populations. *Physical review letters*, 95(23):238701, 2005.

42. Arne Traulsen, Martin A. Nowak, and Jorge M. Pacheco. Stochastic dynamics of invasion and fixation. *Phys. Rev. E*, 74: 011909, 2006.

43. Arne Traulsen, Jorge M. Pacheco, and Lorens A. Imhof. Stochasticity and evolutionary stability. *Phys. Rev. E*, 74:021905, 2006.

44. Vítor V Vasconcelos, Fernando P Santos, Francisco C Santos, and Jorge M Pacheco. Stochastic dynamics through hierarchically embedded markov chains. *Physical review letters*, 118(5):058301, 2017.

45. Carl Veller and Laura K Hayward. Finite-population evolution with rare mutations in asymmetric games. *Journal of Economic Theory*, 162:93–113, 2016.

46. Y. Vorobeychik, M. P. Wellman, and S Singh. Learning payoff functions in infinite games. *Machine Learning*, 67:145–168, 2007.

47. E. Wah, D.R. Hurd, and M.P. Wellman. Strategic market choice: Frequent call markets vs. continuous double auctions for fast and slow traders. In *Proceedings of the Third EAI Conference on Auctions, Market Mechanisms, and Their Applications*, 2015.

48. E. Wah, M. Wright, and M.P. Wellman. Welfare effects of market making in continuous double auctions. *Journal of Artificial Intelligence Research*, 59:613–650, 2017.

49. W. E. Walsh, R. Das, G. Tesauro, and J.O. Kephart. Analyzing complex strategic interactions in multi-agent games. In *AAAI-02 Workshop on Game Theoretic and Decision Theoretic Agents, 2002.*, 2002.

50. W. E. Walsh, D. C. Parkes, and R. Das. Choosing samples to compute heuristic-strategy Nash equilibrium. In *Proceedings of the Fifth Workshop on Agent-Mediated Electronic Commerce*, 2003.

51. X Wang, Y Vorobeychik, and M.P. Wellman. A cloaking mechanism to mitigate market manipulation. In *Proceedings of the 27th International Joint Conference on Artificial Intelligence*, pages 541–547, 2018.

52. Michael P. Wellman. Methods for empirical game-theoretic analysis. In *Proceedings, The Twenty-First National Conference on Artificial Intelligence and the Eighteenth Innovative Applications of Artificial Intelligence Conference, July 16-20, 2006, Boston, Massachusetts, USA*, pages 1552–1556, 2006.

53. M.P. Wellman, T.H. Kim, and Q. Duong. Analyzing incentives for protocol compliance in complex domains: A case study of introduction-based routing. In *Proceedings of the 12th Workshop on the Economics of Information Security*, 2013.

54. M Wright, S. Venkatesan, M. Albenese, and M.P. Wellman. Moving target defense against DDoS attacks: An empirical game-theoretic analysis. In *Proceedings of the Third ACM Workshop on Moving Target Defense*, 2016.

55. H Peyton Young. The evolution of conventions. *Econometrica: Journal of the Econometric Society*, pages 57–84, 1993.
